# Swallowing Rehabilitation With Neuromuscular Electrical Stimulation for Sarcopenic Dysphagia: A Case Report

**DOI:** 10.7759/cureus.59256

**Published:** 2024-04-29

**Authors:** Kazuki Eimoto, Koutatsu Nagai, Yuta Nakao, Yuki Uchiyama, Kazuhisa Domen

**Affiliations:** 1 Department of Rehabilitation, Hyogo Medical University Hospital, Nishinomiya, JPN; 2 Department of Physical Therapy, School of Rehabilitation, Hyogo Medical University, Kobe, JPN; 3 Department of Rehabilitation, Yamato University, Suita, JPN; 4 Department of Rehabilitation Medicine, School of Medicine, Hyogo Medical University, Nishinomiya, JPN

**Keywords:** nmes device, swallowing rehabilitation, strength training, neuromuscular electrical stimulation, sarcopenic dysphagia

## Abstract

Sarcopenic dysphagia is defined as dysphagia caused by sarcopenia of the whole body and swallowing-related muscles. We present a case of sarcopenic dysphagia with improved swallowing function after strength training of swallowing-related muscles using neuromuscular electrical stimulation (NMES). A 78-year-old man was admitted to our hospital with an intraductal papillary mucinous tumor of the pancreatic duct. After admission, the patient developed aspiration pneumonia and was placed on strict bed rest without oral intake, which resulted in progressive malnutrition. At the start of swallowing rehabilitation, he had whole-body sarcopenia, nutritional impairment, and weakness in swallowing-related muscles, with a maximum tongue pressure of 21.4 kPa and an opening force of 5.1 kg. In the videofluoroscopic swallowing study, he aspirated 3 cc of a moderately thick liquid. Consequently, as part of swallowing rehabilitation, strengthening training of swallowing-related muscles with NMES was undertaken for about three weeks. As a result, the maximum tongue pressure and opening force improved to 28.4 kPa and 6.8 kg, respectively. A subsequent videofluoroscopic swallowing study showed no obvious aspiration during any procedure. The patient was discharged on the 86th day on a regular diet. As a component of swallowing rehabilitation, NMES may offer therapeutic benefits for patients with sarcopenic dysphagia.

## Introduction

Sarcopenia has attracted significant attention owing to its association with life expectancy, falls, fractures, and decrement in activities of daily living (ADL) [1]. The etiology of sarcopenia can be categorized as primary or secondary. Primary sarcopenia occurs when there is no obvious cause other than aging, whereas secondary sarcopenia is caused by disease, disuse due to inactivity, or nutritional disorders. Sarcopenia is a potential cause of dysphagia [2]. Sarcopenic dysphagia is defined as dysphagia linked to atrophy of the whole body and swallowing-related muscles, excluding cases in which an obvious disease causes dysphagia [3].

Management strategies for dysphagia induced by sarcopenia include interventions targeting the underlying sarcopenia, swallowing rehabilitation, and nutritional therapy [4]. Neuromuscular electrical stimulation (NMES) is most commonly used in feeding and swallowing training by speech-language pathologists in the United States [5]. However, there are limited reports on swallowing rehabilitation techniques, and the most effective type of swallowing rehabilitation remains unclear.

NMES has emerged as a potent modality for swallowing rehabilitation, demonstrating efficacy in addressing dysphagia resulting from etiologies distinct from sarcopenia [6,7]. In patients with cerebrovascular disease, swallowing rehabilitation incorporating NMES has been shown to shorten oral and pharyngeal transit times, reduce laryngeal penetration and aspiration, and improve oral intake and quality of life [6,7]. The efficacy of NMES has been demonstrated in patients with cerebrovascular disease, and it has recently been recommended as a rehabilitation treatment for sarcopenic dysphagia [8]. However, there are no reports on swallowing rehabilitation using NMES in patients with sarcopenic dysphagia, and its efficacy has not yet been established.

This case study delineates the therapeutic efficacy of combining NMES with swallowing rehabilitation to address sarcopenic dysphagia.

## Case presentation

A 78-year-old man was previously diagnosed with an intraductal papillary mucinous tumor in the pancreatic duct and underwent treatment at another hospital. The patient subsequently presented with recurrent vomiting and was admitted to our hospital for further management. On admission, his anthropometric parameters were as follows: height = 169.7 cm, weight = 54.0 kg, and body mass index (BMI) = 18.7 kg/m2. Prior to admission, the patient was able to walk without assistance, had no decline in ADL, and had not experienced any instances of perceived or suspected dysphagia. Additionally, he had no comorbidities or history of dysphagia, and no medical equipment contraindicated for NMES. At the time of admission, the patient had no oral intake and was receiving peripheral intravenous nutrition (830 kcal/day). Endoscopic ultrasound-guided fine-needle aspiration (EUS-FNA) was performed six days after admission, followed by thoracoscopic gastric jejunal bypass seven days after admission. A liquid diet (900 kcal/day) was initiated three days after the surgery (10 days after admission) and was transitioned to a porridge diet (1650 kcal/day) six days post surgery (13 days after admission). However, on the 10th day after surgery (17th day of admission), the patient experienced an episode of vomiting and developed a fever. Subsequent chest radiography revealed ground-glass opacities and consolidations on the dorsal surfaces of both lungs, suggesting aspiration pneumonia. Consequently, oral intake was discontinued, the patient was placed on strict bed rest, and peripheral parenteral nutrition (400 kcal/day) was initiated. On the 20th day of admission, owing to a worsening respiratory condition, he was transferred to the intensive care unit (ICU) for nasal high-flow therapy. However, his respiratory condition did not improve, and on the 21st day of hospitalization, he underwent endotracheal intubation and was placed on ventilator management. Physical therapy aimed at respiratory and functional rehabilitation was initiated on the 21st day of admission, with a focus on improving respiratory and physical function. After extubation on the 24th day after admission, central venous nutrition (200 kcal/day) was initiated on the 26th day, and a combination of central venous nutrition (1200 kcal/day) and nasal tube feeding (300 kcal/day) was administered on the subsequent day. Swallowing rehabilitation guided by a speech-language-hearing therapist was initiated on the 29th day after admission.

**Figure 1:**
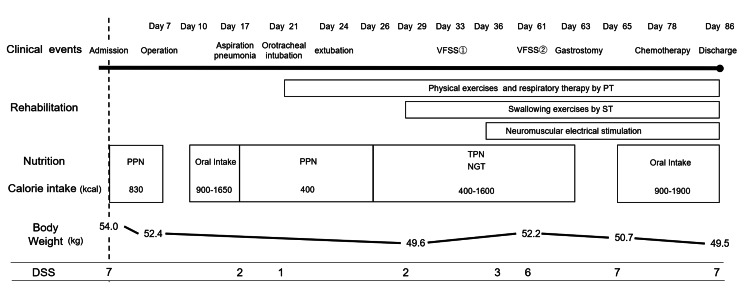
Timeline of the clinical course and treatment. The patient underwent surgery seven days post admission but developed aspiration pneumonia on the 17th day of admission. On the 21st day post admission, the patient was intubated and physical therapy (PT) was initiated. Extubation occurred on the 24th day, and speech therapy (ST) commenced on the 29th day. Neuromuscular electrical stimulation (NMES) was started on the 36th day. The nutritional management during the hospital stay is illustrated in the figure. At the beginning of ST, the patient had severe dysphagia, which improved by the 65th day. The patient was discharged home on the 86th day after receiving chemotherapy. VFSS: videofluoroscopic swallowing study; PT: physical therapy; ST: speech therapy; PPN: peripheral parenteral nutrition; TPN: total parenteral nutrition; NGT: nasogastric tube; DSS: Dysphagia Severity Scale.

Figure 1 shows the timeline of the patient’s clinical course and treatment after admission.

Upon initiation of swallowing rehabilitation, the patient’s weight was recorded as 49.6 kg with a BMI of 17.3 kg/m2. Laboratory findings indicated a serum albumin level of 2.0 g/dL and a C-reactive protein (CRP) level of 3.3 mg/dL. The Mini Nutritional Assessment-Short Form (MNA-SF) score was 4, indicating malnutrition. The patient’s respiratory status required 1 L/min of supplemental oxygen delivered via a nasal cannula, with an oxygen saturation level of 96% at rest. The respiratory rate at rest was 20 breaths per minute, and no obvious abnormal sounds were observed. Chest radiography revealed reduced bilateral pulmonary consolidation, suggesting an improvement in pneumonia. The patient required moderate assistance with ADLs, as reflected by a Functional Independence Measure (FIM) total score of 63. The skeletal muscle mass index (SMI), using a bioelectrical impedance analysis (InBody S10, InBody, Seoul, South Korea), was 6.3 kg/m2, and grip strength, assessed with a Smedley-type digital grip strength meter (GRIP-D, Takei Instrument Industry, Japan), was 18.7 kg on the right and 17.2 kg on the left. Walking speed was not measured because of the difficulty in walking. Based on this information, the patient was diagnosed with sarcopenia according to the Asian Working Group for Sarcopenia (AWGS) 2019 diagnostic criteria [9]. The cognitive function score was 27 out of 30 on the revised Hasegawa’s dementia scale. No significant difference was observed in oral-facial motor skills between the left and right sides. The patient exhibited hoarseness during speech, scoring G1R1B1A1S0 on the GRBAS (grade, roughness, breathiness, asthenia, and strain) scale. However, the symptoms resolved within a few days. Maximum phonation time was 6”14 s. The maximum tongue pressure, measured using a JMS tongue pressure meter (JMS Co., Ltd, Hiroshima, Japan), was 21.4 kPa. The opening force, measured using a jaw-opening stenometer was 5.1 kg. The results of the repetitive saliva swallowing test (RSST) revealed four swallows. Bedside evaluation of swallowing revealed suspected aspiration, as indicated by coughing with 3 mL of a thin liquid and 3 mL of a moderately thick liquid. The cough strength was reasonably maintained. Due to suspected dysphagia, a videofluoroscopic swallowing study (VFSS) was performed on the 33rd day of admission to closely examine swallowing function. After the pharyngeal residue was observed in the pisiform fossa with 3 ml of intermediate-thickened water, aspiration was observed after additional swallowing. During the swallowing of 3 ml of thickened water, a quantitative analysis was conducted using the VFSS images to measure the oral and pharyngeal transit times [6], and the distance of the anterior and upward movement of the hyoid bone [10]. Moreover, the Normalized Residue Ratio Scale (NRRS) [11] was calculated to evaluate the pharyngeal residue in the vallecula and piriform sinuses. Swallow-contrast images were analyzed using the ImageJ image analysis software (National Institutes of Health, Bethesda, MD). The landmarks for the analysis of oral and pharyngeal transit time and anterior/upper hyoid distance were the inferior border of the hyoid bone, the anterior inferior border of the second cervical vertebra, and the anterior inferior border of the fourth cervical vertebra. The test specimens were prepared using 200 ml of barium water diluted to 40% (Baritop, 99% w/w; Kaigen Pharma, Osaka, Japan) and a xanthan-gum-based thickener (Tsururinko Quickly, Clinico, Inc., Tokyo, Japan). Viscosity measurements using the International Dysphagia Diet Standardisation Initiative (IDDSI) Flow Test confirmed that the film was at IDDSI level 3 (moderately thick) [12]. Image analysis revealed that the oral transit time was 0.67 seconds, pharyngeal transit time was 1.2 seconds, anterior hyoid displacement was 7.6 mm, upward displacement was 2.6 mm, Normalized Residue Ratio Scale in the valleculae (NRRSv) was 0, and the Normalized Residue Ratio Scale in the piriform sinus (NRRSp) was 1.37.

Based on the Sarcopenic Dysphagia Diagnosis Flowchart [13], the patient was classified as having "possible sarcopenic dysphagia" and was considered to have dysphagia due to weakness in the swallowing-related muscles caused by sarcopenia.

**Figure 2:**
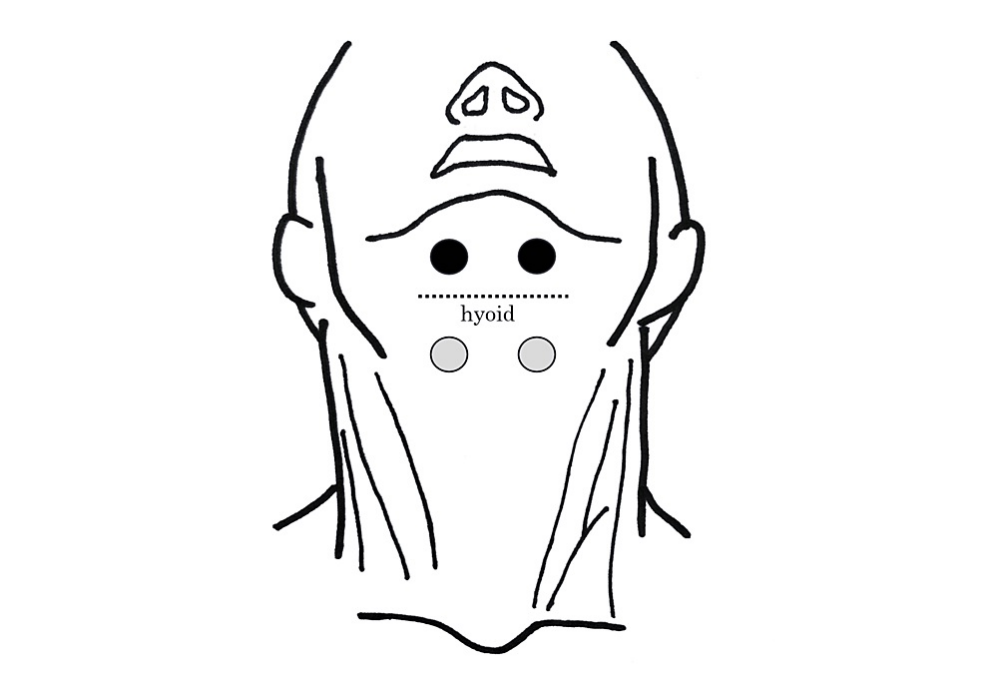
Electrode attachment position.

Beginning on day 36 of hospitalization, indirect swallowing training was performed using the neuromuscular electrical stimulator VitalStim (Chattanooga, Dallas, TX). The NMES electrodes were positioned on the suprahyoid and infrahyoid muscles (Figure 2) [14].

The patient was instructed to shave daily, and sebum was removed with an alcohol pad to reduce electrical resistance at the time of application. The electrical stimulation was set with a frequency of 80 Hz, a pulse duration of 300 μsec, a stimulation time of 55 seconds, a pause of one second, and a ramp-up/down time of two seconds, all within a 60-second cycle. The current intensity was limited to 3 mA on the first day of NMES to reduce mental anxiety, and gradually increased over several days to the maximum (12-15 mA) that the patient could tolerate without experiencing pain [7]. Concurrent with NMES, the patient underwent the following exercises aimed at enhancing the strength of swallowing-related muscles and pharyngeal contractility: (1) tongue-strengthening exercise using a tongue depressor, (2) tongue-hold swallowing, (3) Mendelsohn maneuver, (4) chin push-pull maneuver, and (5) blowing exercise to improve expectoration ability. Swallowing rehabilitation sessions lasted 30-40 minutes each day five days a week, over three weeks. Physical therapy included respiratory rehabilitation, gait training, and muscle strengthening training. Swallowing screening during the second week of training showed no obvious signs of aspiration with 3 ml of intermediately thickened water. Therefore, direct training with 3 ml of intermediately thickened water was initiated during the second week of training.

Our nutrition support team (NST) was consulted for nutritional management. The target nutritional intake was 2219 kcal (calculated as 35 kcal/kg of ideal body weight). We attempted to increase the nutritional intake through a nasogastric tube; however, the patient complained of nausea due to the increased rate of administration associated with higher nutritional intake. Therefore, intake through the nasogastric tube was capped at 800 kcal/day. Consequently, an additional 800 kcal/day was delivered through peripheral intravenous nutrition, increasing the total caloric intake to 1600 kcal/day.

Results

Following three weeks of intervention, laboratory tests revealed a serum albumin level of 3.1 g/dL and a CRP level of 0.3 mg/dL. Body weight increased to 52.1 kg, BMI was 18.1 kg/m2, SMI was 6.9 kg/m2, grip strength showed improvement to 24.2 kg on the right and 23.2 kg on the left, and FIM total score had risen to 79. The maximum tongue pressure was 28.4 kPa, and the opening force was 6.8 kg (Table 1).

**Table 1:** Changes before and after intervention. BMI: body mass index; SMI: skeletal muscle mass index; FIM: Functional Independence Measure; DSS: Dysphagia Severity Scale.

	Pre-intervention (Day 30-32)	Post-intervention (Day 59-61)
Body weight (kg)	49.6	52.1
BMI (kg/㎡)	17.3	18.1
SMI (kg/㎡)	6.3	6.9
Grip strength (kg)	18.7	24.2
FIM total	63	79
FIM-motor	28	44
FIM-cognition	35	35
Maximum tongue pressure (kPa)	21.4	28.4
Jaw-opening force (kg)	5.1	6.8
DSS	2	6

The follow-up VFSS, performed on the 61st day of admission, showed no obvious aspiration in any of the procedures. The VFSS image analysis revealed an oral transit time of 0.3 seconds, pharyngeal transit time of 0.3 seconds, anterior hyoid travel of 10.5 mm, upward travel of 6.1 mm, NRRSv of 0, and NRRSp of 0.54 (Table 2).

**Table 2:** Videofluoroscopic swallowing study analysis. OTT: oral transit time; PTT: pharyngeal transit time; NRRSv: Normalized Residue Ratio Scale vallecula; NRRSp: Normalized Residue Ratio Scale piriform sinuses.

	VFSS 1	VFSS 2
Distance of anterior movement of hyoid (mm)	7.6	10.5
Distance of superior movement of hyoid (mm)	2.6	6.1
OTT (sec)	0.67	0.3
PTT (sec)	1.2	0.3
NRRSv	0	0
NRRSp	1.37	0.54

Due to persistent abdominal problems, a gastrostomy was performed on the 63rd day of admission. The patient was placed on a liquid diet on day 65, which was further modified to a regular diet on day 71. Chemotherapy was initiated on day 78, and after confirming the absence of side effects, the patient was discharged home on day 86.

## Discussion

In the present study, we performed swallowing rehabilitation using NMES to treat dysphagia caused by sarcopenia. Improvement in swallowing function was observed, and the patient was discharged. To the best of our knowledge, no study has elucidated the amalgamation of NMES and swallowing rehabilitation in sarcopenic dysphagia. This is the first report to suggest that swallowing rehabilitation using NMES may be effective in the treatment of feeding and swallowing disorders associated with sarcopenia.

The underlying pathophysiology of dysphagia attributed to sarcopenia is thought to be secondary to systemic diseases, swallowing-related muscle inactivity, and poor nutrition [3]. Prior to admission, the patient exhibited no decline in ADL or episodes of suspected dysphagia. After admission, aspiration pneumonia necessitated bed rest and food abstinence; the patient did not require active nutritional management. Aspiration pneumonia induces the atrophy of respiratory and skeletal muscles throughout the body, including swallowing muscles [15]. Furthermore, another study suggested that patients experiencing aspiration pneumonia coupled with an interval of food deprivation exhibited compromised swallowing function [16]. In the present case, acute inflammatory invasion, inactivity due to rest, and poor nutrition may have led to the rapid progression of secondary sarcopenia, resulting in dysphagia due to the loss of muscle mass.

In the context of swallowing rehabilitation, conventional muscle-strengthening training contracts slow-twitch muscles (type I fibers), while NMES contracts fast-twitch muscles (type II fibers) with thick axons first [17]. Additionally, electrical stimulation of the skeletal muscles in patients with disuse muscle weakness has been shown to accelerate recovery and produce stronger muscle-strengthening effects than conventional training alone [18]. In this case, the use of NMES in addition to conventional swallowing training, such as tongue-strengthening training, tongue-hold swallow, and the Mendelsohn maneuver, may have resulted in muscle-strengthening outcomes. Swallowing rehabilitation using NMES in patients with cerebrovascular disease has been shown to shorten oral and pharyngeal transit times and reduce laryngeal penetration and aspiration [6,7]. In this case of sarcopenic dysphagia, the oral and pharyngeal transit times were shortened, and aspiration was improved, similar to previous reports [6,7], suggesting that swallowing rehabilitation combined with NMES may be effective in patients with sarcopenic dysphagia.

Intervention studies using muscle-strengthening training of swallowing-related muscles have also shown that dysphagia tends to improve more readily when sarcopenia is the cause than when it occurs in other conditions [19]. Given the diagnosis of sarcopenia as the causative factor for dysphagia in this case, a significant improvement in swallowing ability was documented within a short span of three weeks. In conclusion, specialized swallowing rehabilitation should be immediately initiated when dysphagia associated with sarcopenia is suspected.

The therapeutic strategy for sarcopenic dysphagia depends on the underlying etiology of systemic sarcopenia. Therefore, a comprehensive approach is desirable [20]. Specifically, when aging is identified as a precipitating factor for systemic sarcopenia, interventions should include strength training and supplementation of protein and branched-chain amino acids. In cases of inactivity, early weaning and oral intake are recommended. Nutritional optimization is of paramount importance, considering the following equation: daily energy requirement = daily energy expenditure + energy stores (ranging from 200-750 kcal/day). If a disease is the cause, the underlying disease should be treated. In the present case, besides treating pneumonia, the cause of systemic sarcopenia, physical therapy to improve physical function, and strength training of swallowing-related muscles using NMES were performed to improve swallowing function. Although the patient was unable to meet the initial target caloric intake, nutritional management was provided as much as possible with support from the NST. This comprehensive multidisciplinary approach, followed by swallowing rehabilitation using NMES, may have contributed to the improvement in swallowing function.

This study has several limitations. First, because this was a case report, the results cannot be generalized. Second, strict nutritional management was not implemented. While our goal was to manage this patient at the target nutritional level of 2219 kcal, calculated at 35 kcal/kg of ideal body weight, the patient was unable to undergo aggressive nutritional therapy aimed at weight gain because of residual disease. However, even when aggressive nutritional therapy is not feasible, swallowing rehabilitation using NMES can improve dysphagia.

## Conclusions

There are few reports on swallowing rehabilitation for patients with sarcopenic dysphagia, and rehabilitation methods have not yet been established. This report suggests that feeding and swallowing rehabilitation using NMES may be effective for feeding and swallowing disorders associated with sarcopenia. This finding will guide future studies investigating effective rehabilitation treatment for sarcopenia-associated feeding and swallowing disorders.
